# The Antioxidant Peptide Salamandrin-I: First Bioactive Peptide Identified from Skin Secretion of Salamandra Genus (*Salamandra salamandra*)

**DOI:** 10.3390/biom10040512

**Published:** 2020-03-27

**Authors:** Alexandra Plácido, João Bueno, Eder A. Barbosa, Daniel C. Moreira, Jhones do Nascimento Dias, Wanessa Felix Cabral, Patrícia Albuquerque, Lucinda J. Bessa, Jaime Freitas, Selma A. S. Kuckelhaus, Filipe C. D. A. Lima, Augusto Batagin-Neto, Guilherme D. Brand, João B. Relvas, José Roberto S. A. Leite, Peter Eaton

**Affiliations:** 1 LAQV/REQUIMTE, Department of Chemistry and Biochemistry, Faculty of Sciences, University of Porto, 4169-007 Porto, Portugal; lucinda.bessa@fc.up.pt (L.J.B.); jrsaleite@gmail.com (J.R.S.A.L.); peter.eaton@fc.up.pt (P.E.); 2 Institute for Research and Innovation in Health (i3S) and Institute for Molecular and Cell Biology (IBMC), 4200-135 Porto, Portugal; jrelvas@ibmc.up.pt; 3 Center for Research in Applied Morphology and Immunology, NuPMIA, University of Brasilia, Brasilia, DF 70910-900, Brazil; joaobueno70@gmail.com (J.B.); moreiradc@unb.br (D.C.M.); wanessa.wfc@gmail.com (W.F.C.); selmask@gmail.com (S.A.S.K.); 4 Laboratory for the Synthesis and Analysis of Biomolecules (LSAB), Institute of Chemistry, University of Brasília, Brasília, DF 70910-900, Brazil; bioederr@gmail.com (E.A.B.); gdbrand@unb.br (G.D.B.); 5 Laboratory of Molecular Biology of Fungal Pathogens, Department of Cell Biology, Institute of Biological Sciences, University of Brasília, Brasília, DF 70910-900, Brazil; jhonesnd@gmail.com (J.d.N.D.); palbuquerque@unb.br (P.A.); 6 Institute for Research and Innovation in Health (i3S) and National Institute of Biomedical Engineering (INEB), 4200-135 Porto, Portugal; jaime.freitas@i3s.up.pt; 7 Federal Institute of Education, Science and Technology of São Paulo, Campus Matão, Matão, SP 15991-502, Brazil; fdlima@ifsp.edu.br; 8 São Paulo State University (UNESP), Campus of Itapeva, Itapeva, SP 18409-010, Brazil; a.batagin@unesp.br

**Keywords:** antioxidant peptides, *Salamandra salamandra*, portuguese biodiversity, bioactive molecules

## Abstract

Amphibian skin is a multifunctional organ that plays key roles in defense, breathing, and water balance. In this study, skin secretion samples of the fire salamander (*Salamandra salamandra*) were separated using RP-HPLC and de novo sequenced using MALDI-TOF MS/MS. Next, we used an in silico platform to screen antioxidant molecules in the framework of density functional theory. One of the identified peptides, salamandrin-I, [M + H]^+^ = 1406.6 Da, was selected for solid-phase synthesis; it showed free radical scavenging activity against DPPH and ABTS radicals. Salamandrin-I did not show antimicrobial activity against Gram-positive and -negative bacteria. In vitro assays using human microglia and red blood cells showed that salamandrin-I has no cytotoxicity up to the concentration of 100 µM. In addition, in vivo toxicity tests on *Galleria mellonella* larvae resulted in no mortality at 20 and 40 mg/kg. Antioxidant peptides derived from natural sources are increasingly attracting interest. Among several applications, these peptides, such as salamandrin-I, can be used as templates in the design of novel antioxidant molecules that may contribute to devising strategies for more effective control of neurological disease.

## 1. Introduction

Amphibians are widely distributed across numerous habitats worldwide. Most amphibians produce secretions that are released onto their skin when myocytes surrounding the granular glands contract in response to stimuli promoted by the sympathetic nervous system [1]. Amphibian secretions have been used for centuries as ethno-pharmaceutical drugs in Asia and in the Americas for the treatment of several diseases, such as hemorrhages, allergies, inflammations and infections, and more recently have been considered as a prolific source of potential drug leads [2,3]. Among the molecules constituting amphibian skin secretions, a myriad of peptides have gained attention due to their wide range of biological activities. These peptides are thought to play various roles, either in the regulation of physiological functions of the skin, or in the defense against predators and microorganisms [4]. Additionally, many of the amphibian peptides belong to families of bioactive peptides that have mammalian counterparts, such as caerulein, bombesin/gastrin-releasing peptide, and exendin-4/glucagon-like peptide-1 [5], or antimicrobial peptides.

Amphibian skin is subject to several threats, such as both endogenous and exogenous sources of oxidizing agents, which at a physiological level cause oxidative stress response. Yang et al. [6] showed that amphibians living in high-altitude environments have developed behavioral and physiological adaptations to the low temperatures, as well as the reduced oxygen availability characteristic of their local environment. Accordingly, antioxidant peptides (AOPs) have been isolated and characterized from the skin secretions of some frogs [7,8,9]. Recently, Barbosa et al. [10] identified a peptide called antioxidin-I from the skin secretions of four South American frog species. Despite having low antioxidant potential against the widely used ABTS and DPPH radical models (2,2’-azino-bis(3-ethylbenzothiazoline-6-sulfonic acid) and di(phenyl)-(2,4,6-trinitrophenyl)iminoazanium, respectively), antioxidin-I strongly inhibited intracellular ROS formation in human microglia exposed to hypoxia, indicating that antioxidin-I may be useful in strategies aiming at controlling oxidative stress in neurological disease, which should be subjected to further research [10].

Salamanders belong to the order of the Urodela, which corresponds to 9% of amphibians. They present a wide variety of antipredator mechanisms, including toxins and noxious or adhesive skin secretions. Fire salamanders, *Salamandra salamandra* (Linnaeus, 1758), are widespread in southern and central Europe [11], normally at altitudes between 250 and 1000 m, although in the Balkans and in Spain, they are commonly found at higher altitudes [12]. The poison glands of the fire salamanders are concentrated in certain areas of the body, especially around the head and the dorsal skin surface. The colored portions of the animals′ skin often coincide with these glands, representing an aposematic signal. Compounds in the skin secretions may be effective against bacterial and fungal infections of the epidermis; some are potentially dangerous to humans [13]. For instance, the fire salamander’s primary steroidal alkaloid toxin, samandarin, causes strong muscle convulsions and hypertension combined with hyperventilation in all vertebrates. The skin secretions of Urodela amphibians are still largely unexplored with respect to bioactive molecules, including peptides, especially when compared to the vast amount of data available on the skin secretions of anurans. Steroids, biogenic amines, and alkaloid molecules have been identified in European fire salamander, however their pharmacological potential remains to be discovered [14]. To the best of our knowledge, this is the first work describing a bioactive peptide isolated from skin secretion of the Salamandra genus.

In this work, we report the discovery of salamandrin-I peptide (FAVWGCADYRGY-NH_2_), identified from the skin secretions of *S. Salamandra*. This novel antioxidant compound is the first bioactive peptide isolated from the European fire salamander. Bioprospection, synthesis, purification, and structural, computational and biological analyses were performed, including antibacterial activity and toxicological assessment.

## 2. Materials and Methods

### 2.1. Biological Samples

**Figure 1:**
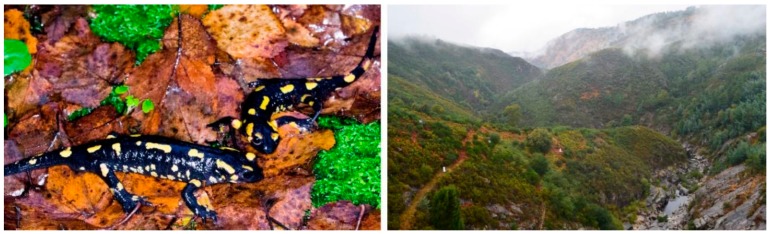
Adult specimens of *Salamandra salamandra* (**left**) (Photo: Peter Eaton). Peneda-Gerês National Park, habitat of *S. Salamandra* (**right**) (Photo: José Roberto Leite).

Adult *Salamandra* specimens were manually captured in the autumn in Peneda-Gerês National Park, located northeast of the Minho region, Portugal, under the license No. 364/2018 CAPT/ICNF (conceded by the ICNF, Institute for Conservation of Nature and Forests, Portugal) (https://youtu.be/o6c01HLF3mM) (Figure 1). The skin secretions were obtained from *S. salamandra* using gentle electrical stimulation (9 V) over the moistened dorsal skin surface for a few seconds and collected into tubes using Milli-Q water. Afterwards, the resultant secretions were filtered using MF-Millipore^TM^ membrane filters with 0.22 μm pore size, frozen and lyophilized (SP Scientific Virtis BTP-9EL00X, New York, NY, USA).

### 2.2. Purification and Characterization

First, 1 mg of dry secretion was dissolved in 500 μL of Milli-Q water and separated using an LC-20 CE model HPLC system (Shimadzu Co., Kyoto, Japan), using a Vydac C18 reverse phase column (218 TP). Fractions were eluted with a linear gradient of 0.1% (*v*/*v*) Trifluoroacetic Acid (TFA)/acetonitrile ranging from 5% to 60% over 60 min and 75%–95% over 5 min at a flow rate of 1 mL/min. Absorbance was monitored at 216 and 280 nm, and fractions were hand collected in tubes and dried under vacuum centrifugation (Thermo Electron Corporation, RVT400-115, Waltham, MA, USA). Subsequently, dried fractions were re-dissolved in Milli-Q water in a range from 10 to 100 μL, which was adjusted according to the UV absorbance obtained for each fraction. In order to determine the molecular mass of the peptides, Matrix Assisted Laser Desorption Ionization Time of Flight Mass Spectrometry (MALDI TOF MS) analyses using an UltraFlex III mass spectrometer (Bruker Daltonics, Bremen, Germany) in the positive reflected mode and controlled using FlexControl software were performed. For this purpose, 1 μL aliquots of the chromatographic fractions were dissolved in α-cyano-4-hydroxicinamic acid matrix solution (1:3, *v*/*v*) and then applied on the MALDI plate. The ions of interest were fragmented in the LIFT mode (MS/MS) for de novo sequencing. Before each analysis, the spectrometer was calibrated using a mixture of peptides, according to the manufacturer’s instructions. The spectra were analyzed manually using FlexAnalysis software (Bruker Daltonics, Bremen, Germany), and a prediction of isotopic abundance was realized using Compass IsotopePattern software (Bruker Daltonics, Bremen, Germany) [10].

### 2.3. Peptide Synthesis

The peptide salamandrin-I (FAVWGCADYRGY-NH_2_) was chemically synthesized using the F-moc/t-butyl method [15]. Briefly, Rink amide resin (Novabiochem, San Diego, CA, USA) was swollen for 20 min with dichloromethane and submitted to repeated cycles of Fmoc deprotection using 20% 4-methylpiperidine in N,N-dimethylformamide (DMF) and peptide chain elongation using 4 equivalents of Fmoc-amino acids, N′-diisopropylcarbodiimide, and OxymaPure^®^, and also using DMF as solvent. After the completion of the synthesis, the N-terminal Fmoc protecting group was removed and peptide cleavage from resin was performed using a cocktail consisting of TFA:thioanisole:water:phenol:1,2-ethanedithiol 82.5:5:5:5:2.5 (*v*/*v*) under agitation for 1.5 h. Synthetic products were freeze-dried and submitted to MALDI MS analysis using the previously described equipment and methodology. Peptide purification was performed using RP-HPLC with a Jupiter COG 4055-P0 preparative column coupled to a Shimadzu LC-20 CE chromatographer programmed to submit samples to a linear gradient of 0.1% (*v*/*v*) TFA/acetonitrile at a flow rate of 10 mL/min. Further details on the methodology used for peptide purification and mass spectrometric analysis to confirm purity and primary structure can be found elsewhere [16]. For all experiments, the synthesized and isolated salamandrin-I was first solubilized in DMSO before dilution in the appropriate medium for each assay.

### 2.4. Structural Studies

#### 2.4.1. Sequence Analysis

The peptide sequence was compared with antimicrobial peptides deposited at the antimicrobial peptide database [17]. Physical and chemical parameters (MW, theoretical pI, instability index, aliphatic index, and grand average of hydropathicity, GRAVY) were calculated using the ProtParam website, while hydrophobic moments (μH) and helix wheel projections were determined using the HeliQuest program [18]. Peptide concentration was measured by using the following equation: [peptide concentration] mg/mL = (A_280_ × DF × Mw)/ε, where A_280_ is the absorbance of the peptide solution at 280 nm in a 1 cm cell, DF is the dilution factor, Mw is the molecular weight of the peptide, and ε is the molar extinction coefficient of tryptophan or tyrosine at 280 nm [19].

#### 2.4.2. Circular Dichroism

The secondary structure of salamandrin-I was assessed using circular dichroism (CD) spectroscopy in the far UV region using a Jasco J-815 CD Spectropolarimeter (Jasco Corp., Tokyo, Japan), as previously reported [20,21]. Briefly, the measurements were carried out under a nitrogen gas flow of 8 L/h at 20 °C. Spectra were obtained between 190 and 260 nm, using a 100 mm cell path length. Salamandrin-I was diluted at 100 μM final concentration and 2,2,2-trifluoethanol (TFE) at *v*/*v* rations of 10%, 20%, and 40% in Milli-Q water. These experiments were performed at 37 °C and a scan speed of 50 nm/min, a response time of 1 s, and a bandwidth of 1 nm were used. The spectra were converted to molar ellipticity per residue, as previously reported [20,21].

### 2.5. In Vitro Radical Scavenging Assays

Free radical scavenging activity was assessed in vitro using two chemical-based assays, ABTS and DPPH assays. For both assays, salamandrin-I was diluted in PBS containing 27% (*v*/*v*) DMSO to prepare the stock solution at 1.5 mg/mL. The stock solution was diluted to several concentrations (0.03125–0.2500 mg/mL) using PBS prior to the assays. Free radical scavenging activity was determined using the 2,2-azinobis-3-ethylbenzothiazoline-6-sulphonic acid (ABTS^+^) method as described by Gião et al. [22] First, to oxidize the colorless ABTS to the blue-green ABTS^+^ radical cation, ABTS (7 mM) was mixed with ammonium persulfate (2.45 mM) and kept for 12–16 h at room temperature in the dark. Then, the ABTS^+^ solution was diluted with water to an absorbance of 0.70 at 734 nm (Shimadzu 1240 UV–visible spectrophotometer, Kyoto, Japan). Aliquots (10 µL) of salamandrin-I, Trolox, and glutathione (diluted in PBS and used for comparison purposes) were mixed with 190 µL of ABTS^+^ solution and the absorbance at 734 nm was read at 6 min after the onset of the reaction. The decrease (%) in absorbance at 734 nm, which corresponds to the concentration of ABTS^+^, caused by salamandrin-I and glutathione was compared with that of a standard curve built with different concentrations (2–64 µg/mL) of Trolox (6-hydroxy-2,5,7,8-tetramethylchroman-2-carboxylic acid). Results were expressed as mg of Trolox equivalents/mg peptide.

For the DPPH assay, a stock solution of 1,1-diphenyl-2-picrylhydrazyl (DPPH) was prepared in ethanol at 60 μM. Then, the DPPH solution was adjusted with ethanol to achieve an absorbance of 0.7 at 515 nm (Shimadzu 1240 UV-visible spectrophotometer, Kyoto, Japan). Salamandrin and glutathione were dissolved as described for the ABTS assay. Aliquots (20 µL) of salamandrin-I, Trolox, and glutathione (diluted in PBS and used for comparison purposes) were mixed with 180 µL of DPPH ethanolic solution and the absorbance at 515 nm was read at 30 min after the onset of the reaction [23]. The decrease (%) in absorbance at 515 nm, which corresponds to the concentration of DPPH, caused by salamandrin-I and glutathione was compared with that of a standard curve built with different concentrations (2–64 µg/mL) of Trolox (6-hydroxy-2,5,7,8-tetramethylchroman-2-carboxylic acid). Results were expressed as mg of Trolox equivalents/mg peptide.

### 2.6. In Silico Antioxidant Studies

The peptide was designed using Avogadro software [24], taking into account the amide modification on the C-terminal. In order to better reproduce structural features of the salamandrin-I peptide, a preliminary conformational search was conducted via molecular dynamics (MD) calculations at a high temperature [25]. For this purpose, the molecule was placed in contact with a thermal reservoir at 1000 K, and 50 distinct conformations were stored during the dynamics for subsequent geometry optimization. MD conformational searches were conducted using AMBER force field [26] with the aid of the Gabedit computational package [27]. Preliminary geometry optimizations were conducted for all the conformers in a Hartree–Fock (HF) approach using the semiempirical Hamiltonian PM6 [28] implemented in the MOPAC2016 computational package [29]. The geometry of the most stable conformer was then fully optimized in the framework of Kohn–Sham density functional theory (KS-DFT) using Becke’s three-parameter hybrid exchange functional with the Lee–Yang–Parr correlation functional (B3LYP) [30,31,32,33], and 6-31G(d,p) basis set on all the atoms. Glutathione and Trolox geometries were directly optimized in a KS-DFT/B3LYP/6-31G(d,p) considering the 3D geometries available in the PubChem database as input [34]. For a finite system, such as a molecule, once the number of electrons is a discrete variable, there is a dependence of the electronic density (and chemical reactivity) with the removal/inclusion of the electrons in the system that can be understood in terms of how the atomic charge reorganizes, which is defined as the condensed-to-atom Fukui indexes (CAFI) [35,36]. These descriptors define three different types of reactions, towards nucleophiles (*f_k_^+^*), electrophiles (*f_k_^−^*), and free radicals (*f_k_^0^*), and are expressed in the following equations:

*f*_k_^+^ = q_k_ (N + 1) – q_k_ (N); (1)

*f*_k_^−^ = q_k_ (N) – q_k_ (N – 1); (2)

*f*_k_^0^ = 1/2 [q_k_ (N + 1) – q_k_ (N − 1)]; (3)

where q_k_ (N + 1), q_k_ (N), and q_k_ (N − 1) represent electronic population of the *k*-th atom of the compound in its anionic (with N + 1 electrons), neutral (with N electrons), and cationic (with N − 1 electrons) configurations, respectively (without geometry relaxation). The CAFIs were calculated using the same level of theory, functional, and basis set employed in the salamandrin-I optimizations. The Hirshfeld partition charge method was employed to avoid negative CAFI values [37,38]. The local softness (s_k_^+^, s_k_^−^, and s_k_^0^) was estimated from the CAFI values by multiplying them by the global softness of the compounds [35,36]. DFT-based calculations were conducted with the aid of the Gaussian 09 computational package [39]. 

Finally, to compare the antioxidant properties of salamandrin-I, glutathione, Trolox, and other compounds, the electron acceptance (Ra) and electron donation (Rd) indexes proposed by Martínez et al. [40] were calculated and evaluated in terms of donor-acceptor map (DAM) [41].

### 2.7. Antibacterial Assays

The antibacterial activity of peptide salamandrin-I was assessed using both the disk diffusion method and the microdilution method, performed according to the Clinical and Laboratory Standards Institute (CLSI) guidelines [42,43]; our bacterial strains were used in both assays (*Escherichia coli* ATCC 25922, *Pseudomonas aeruginosa* ATCC 27853, *Staphylococcus aureus* ATCC 25923 and *Enterococcus faecalis* ATCC 29212).

Each bacterial inoculum (approximately 10^8^ CFU/mL) was spread on Mueller–Hinton agar plates. Subsequently, blank paper discs (6mm in diameter) were placed on the agar surface and impregnated with 15 μL of a 10 mg/mL stock solution in DMSO of the peptide or with 100% (*v*/*v*) DMSO (used as a negative control). A commercial antibiotic disk, Cefepime (FEP, 30 µg, Oxoid, Thermo Fisher Scientific, Basingstoke, England), was used as positive control. Plates were kept for 2 h at room temperature and then incubated at 37 °C for 18–24 h. Inhibition zones around the disks were compared with the control (DMSO) and measured (mm).

The minimum inhibitory concentration (MIC) of peptide salamandrin-I was determined following the exact procedure as described by Bessa et al. [44]. The peptide was tested in the concentration range of 1–1024 μg/mL against each strain.

### 2.8. Toxicity Studies

#### 2.8.1. Cytotoxicity Assessment

The hemolytic activity of salamandrin-I was tested using human red blood cells (RBCs)/O+, as previously described [45], with some modifications. The isolation of immune cells from healthy blood donors was approved by the *Centro Hospitalar Universitário São João* Ethics Committee (protocol 90/19), after each donor informed consent collection. The i3S researchers involved in the project from Glial Cell Biology lab are accredited by FELASA (B and C) and all projects in i3S follow the rules and recommendations of FELASA. Briefly, RBCs were collected in EDTA (1.8 mg/mL), washed 4 times with PBS (pH 7.4), and pellets were resuspended and diluted with the same solution. The RBC suspension was added to an equal volume of each peptide solution at different concentrations (0–200 µM). The mixtures were incubated for 1 h at 37 °C and then centrifuged at 2400 *g* for 5 min. The supernatants were removed, and the value of absorbance (A) at 492 nm was measured. Maximum hemolysis was determined by adding 0.1% Triton X-100 (*v*/*v*) to a sample of cells, and PBS was used as negative hemolysis control. The hemolysis percentage was calculated as follows:

[(A_peptide_ − A_PBS_)/(A_Triton_ – A_PBS_)] × 100. (4)

The cytotoxicity of salamandrin-I was also assessed using the HMC3 (human microglial clone 3 cell line) that was established in 1995, through SV40-dependent immortalization of human embryonic microglial cells. Cell viability was determined by measuring total cellular metabolic activity using the reduction of resazurin to the fluorescent resorufin. Briefly, following 24 h exposure to salamandrin-I, 8 μL of a 400 μM resazurin solution was added to each well. After four hours of incubation in the dark (37 °C; 95% air, 5% CO_2_), fluorescence was measured at λ_excitation_ = 530 nm and λ_emission_ = 590 nm. All exposures were performed in triplicate and every assay was repeated in triplicate [46]. Statistical analysis and graph construction was performed using GraphPad Prism 6.0 software [47]. Results were tested for normality using the Shapiro–Wilk normality test, and as a Gaussian distribution was observed, an ordinary one-way ANOVA test was applied.

#### 2.8.2. In Silico Toxicity

Analyses were performed with the aid of the on-line platform pkCSM [48,49].

#### 2.8.3. In Vivo Toxicology

Sixteen instar *Galleria mellonella* larvae (250–300 mg) were randomly selected to compose groups and used for toxicity tests, according to Ignasiak and Maxwell [50]. In each group, 10 μL of different doses of salamandrin-I peptide (40, 20, and 10 mg/kg) were injected. A control with PBS and 6% DMSO was performed under the same conditions as the peptides. After treatment, larvae were incubated at 37 °C and the number of deaths counted daily for seven days. The assay was performed twice, and the results were evaluated using GraphPad Prism 6.0 software. After seven days, a histological analysis of the PBS, DMSO, and salamandrin-I groups at 40 mg/kg was performed with Heidenhain’s aniline blue stain staining to identify histological deformations [51,52]. Histological images were taken using an inverted microscope (Axio Observer Z1-Carl Zeiss Microscopy, New York, NY, USA). All groups were counted equally and daily until the seventh day. The samples were diluted in DMSO, and the doses were adjusted with PBS.

## 3. Results and Discussion

### 3.1. Purification and Characterization

**Figure 2:**
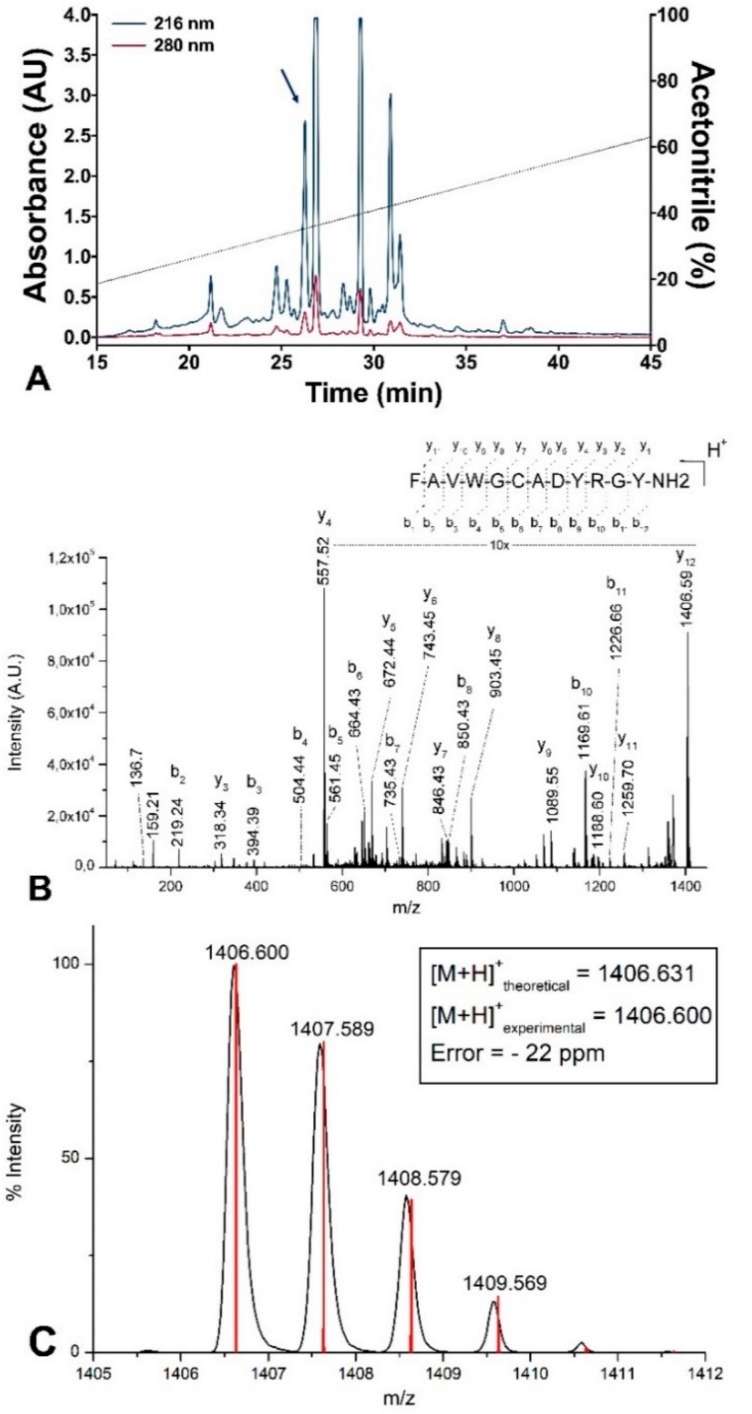
(**A**) Reversed-phase HPLC chromatogram of the crude extract from *S. salamandra* skin secretions. Sample absorbance was monitored at 216 (blue line) and 280 nm (red line). Fraction containing peak at retention time 26 min (blue arrow) corresponds to salamandrin I peptide. (**B**) MS/MS spectrum of salamandrin-I, [M + H]^+^ = 1406.6 Da, acquired in an Ultraflex III MALDI TOF MS; amino acid sequence determined: FAVWGCADYRGY-NH_2_. (**C**) MS spectrum showing isotopic profile and predicted isotope abundance (red line) of salamandrin-I peptide. Square shows the calculated error.

In this study, lyophilized skin secretions of *S. salamandra* were fractionated using C_18_ RP-HPLC, yielding a profile of approximately 20 fractions (Figure 2A).

Subsequently, all aliquots were analyzed using MALDI TOF MS, and selected ions were fragmented for de novo sequencing. Among the identified peptides, salamandrin-I ([M + H]^+^ = 1406.6 Da) was selected and manual interpretation of its MS/MS spectrum allowed the identification of the amino acid sequence FAVWGCADYRGY-NH_2_, showing the presence of a post-translational modification at the C-terminal (carboxiamide) (Figure 2B). The prediction of isotope abundance of salamandrin-I peptide corroborates the amino acid sequence obtained by MS/MS experiments, as well as the presence of C-terminal amidation (Figure 2C). The choice of salamandrin-I for further chemical synthesis and biological testing was based on three points: 1) molecular weight below 2 kDa, 2) amino acids in its sequence, which are related to potential electron transfer, such as W and Y, and 3) chemical reactivity characteristics, according to in silico analysis (which will be discussed later in this section).

The primary structure of salamandrin-I was compared to other molecules in appropriate databases (e.g., PepBank), and the result was that this is an unprecedented peptide with unknown biological function. Therefore, the sequencing of salamandrin-I resulted in the first bioactive peptide identified in *S. salamandra* skin secretions. However, it has 100% identity with the N-terminal region of CFBD-1 (Figure S1), a polypeptide with moderate antimicrobial activity recently found in the skin of the Chinese salamander (*Cynops fudingensis*), as determined by Edman degradation and cDNA sequencing (Table 1) [53].

The peptide CFBD-1 was classified as a β-defensin based on its sequence similarity with β-defensins from other vertebrates [53]. As far as we know, CFBD-1 is the first β-defensin antimicrobial peptide from a salamander. In the skin secretions of *S. salamandra* some un-sequenced ions with a mass of 4 kDa were identified using MALDI TOF MS (data not shown), but the chance of FAVWGCADYRGY-NH_2_ being a truncated peptide of the defensin class of polypeptides seems remote due to the presence of the amidated C-terminal. However, as the mechanism governing the production of bioactive peptides constituting amphibian skin tissue are not entirely understood, some experiments involving mRNA sequencing of salamandrin-I precursor can confirm our findings. It is important to mention that, if this is the case, antimicrobial peptides can undergo proteolysis after they are exuded from granular glands and, consequently, can acquire another biological activity.

**Figure 3:**
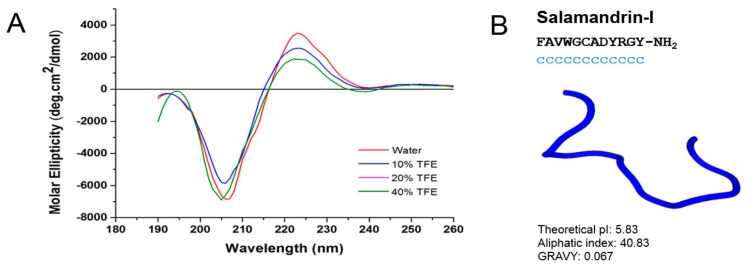
Structural analysis of salamandrin-I peptide. (**A**) Circular dichroism of peptide in aqueous solution and 10%, 20%, and 40% of 2,2,2-trifluoethanol (TFE) solutions (red, blue, pink, and green, respectively). (**B**) Theoretical salamandrin-I 3D structure prediction (c, random coil representation/GRAVY, Grand Average of Hydropathy).

The secondary structure of salamandrin-I was assessed experimentally using CD analysis (Figure 3A). Salamandrin-I presents low molar ellipticity, and a minimum at around 205 nm, demonstrating a random coil conformation both in water and in TFE (Figure 3A). Interestingly, antioxidin-I [10], an antioxidant peptide, also showed a random coil structure both in water and 10%, 20%, and 40% of TFE. Theoretical 3D structure studies performed in silico using PEP-FOLD, were consistent with CD results [54], where it was observed that the peptide has a random coil conformation (Figure 3B). The in silico analysis also indicates that that all amino acid residues are in the random coil conformation in aqueous solution, that is, none of them showed any tendency to form defined secondary structures. However, although the pI and average hydropathy (GRAVY) parameters are similar for salamandrin-I and antioxidin-I, the aliphatic index of salamandrin-I is much higher leading to low solubility in water. Although these results are intriguing, they cannot conclude on cell penetrability alone; now we are going to direct experiments with fluorescent markers and radioisotopes to try to understand if this peptide has the capacity of cell penetrator.

### 3.2. Antimicrobial Activity

The structural similarity of salamandrin-I with the β-defensin N-terminal region impelled us to perform antimicrobial assays against Gram-negative bacteria (*E. coli* ATCC 25922 and *P. aeruginosa* ATCC 27853) and Gram-positive bacteria (*S. aureus* ATCC 25923 and *E. faecalis* ATCC 29212). However, salamandrin-I did not show antibacterial activity against any of the bacterial species tested. Using the disk diffusion method, no zone of growth inhibition around the peptide-impregnated disk was observed. Similarly, no MIC could be obtained in the range of concentrations tested (MIC >1024 µg/mL). Afterwards, salamandrin-I was analyzed in the APD database (the antimicrobial peptide database, http://aps.unmc.edu/AP/), and no peptide with structural similarity was found in this databank. This database focuses on natural antimicrobial peptides (AMPs) with defined sequences and activity. The APD databank includes a total of 2619 antimicrobial peptides with 261 bacteriocins from bacteria, 4 from archaea, 7 from protists, 13 from fungi, 321 from plants, and 1972 animal host defense peptides. This search is consistent with our experimental antibacterial data. Furthermore, in CD studies a random coil conformation is evident for salamandrin-I regardless of solvent polarity, lacking the alpha-helix secondary structure typically described for AMPs. This behavior was observed with antioxidin-I peptide, which also showed no antimicrobial activity, despite having antioxidant activity. It is worth mentioning that this is not always the case for AOPs. Indeed, some antioxidant peptides (AOPs) may have more than one activity, including antimicrobial properties. For instance, some pleurains (e.g., Pleurain-A1), have antioxidant activity, and in their sequences, the presence of three Lys residues confers them a bactericidal effect [7].

### 3.3. In Vitro Radical Scavenging

The DPPH and ABTS^+^ radicals are commonly used to evaluate in vitro potential antioxidant activity. The ABTS system is commonly correlated with the DPPH system because these two radicals have similar hydrogen/electron donation characteristics. Antioxidant candidates under study react with DPPH and ABTS^+^ and convert the colored stable free radicals into colorless non-radical compounds. The amount of reduced DPPH and ABTS^+^ can thus be quantified by measuring the decrease in absorbance at 515 and 734 nm, respectively [54].

The ability of salamandrin-I and glutathione, a peptide used as positive control, to scavenge radicals in vitro was evaluated using two different systems (ABTS and DPPH) (Table 2). Glutathione is known as the most abundant low molecular weight thiol in eukaryotic cells, where it participates in many signaling pathways and functions as a redox buffer in concert with glutathione-dependent enzymes [54]. Glutathione was chosen for comparison for also being a peptide natural product (PNP) with a well-described antioxidant role.

The results indicate that both salamandrin-I and glutathione have free radical scavenging activities, mainly for the ABTS radical. In the literature, the identification of bioactive peptides with antioxidant activity for ABTS and little or no activity for DPPH has been recurrent, such as plaurain-A1 [6] and antioxidin-I [10]. Although the mechanism is not yet understood, due to the resonant structure of the ABTS active site, a more effective interaction of the reactive salamandrin-I groups is expected.

Many antioxidant peptides with variable structures have been recently isolated, including antioxidin-RP1 and antioxidin-I [10]. Such activity was attributed to the presence of critical residues of methionine, cysteine, tyrosine, and proline [6]. In the specific case of salamandrin-I, tryptophan, cysteine, and tyrosine amino acid residues may be related to its antioxidant activity. Regardless, such in vitro assays are highly limited, and the interpretation of their potential activity in vivo should be corroborated by other methods. To better understand the structural relationship of these molecules and to be able to design and perform future cell-based experiments, we will discuss below reactivity parameters coming from in silico studies.

### 3.4. In Silico Antioxidant Studies

The conformational study of the peptide was conducted via molecular mechanics simulations at high temperature (1000 K) to investigate varied conformers. From these studies no particular secondary structure was found, reinforcing the PEP-FOLD results.

**Figure 4:**
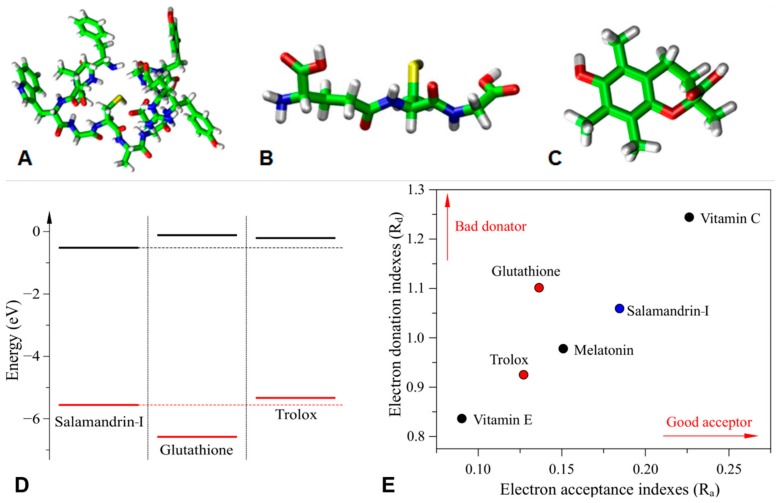
Density functional theory (DFT) optimized structures of (**A**) salamandrin-I, (**B**) glutathione, and (**C**) trolox. Atomic colors: C (green), O (red), S (yellow), N (blue) and H (white). (**D**) Frontier energy level alignments between salamandrin-I, glutathione, and Trolox (highest occupied molecular orbital (HOMO): red and lowest unoccupied molecular orbital (LUMO): black). (**E**) Donor-acceptor map for varied antioxidants. The reference values obtained for melatonin, vitamin E and vitamin C were obtained from [41].

In order to understand the antioxidant activity-structure relationship of salamandrin-I, electronic properties of the peptide were investigated and compared with glutathione and Trolox, whose structures are presented in Figure 4A–C. Figure 4D,E show the relative position of the frontier orbitals (HOMO: highest occupied molecular orbital and LUMO: lowest unoccupied molecular orbital), as well as the DAM associated with these compounds and other common antioxidants [55].

Note that salamandrin-I, glutathione, and Trolox present similar frontier energy levels, which suggests that these compounds have analogue electron acceptor/donor properties. The highest dissimilarity is associated with the HOMO level of glutathione. In general, good electron donors present higher HOMO levels (which results in lower Rd values), while lower LUMOs are associated with good acceptors (i.e., high Ra values). This information is summarized in Figure 4E, which highlights the interesting trend between donor/acceptor properties of samalandrin-I compared to the other compounds (good acceptor and moderate donator).

**Figure 5:**
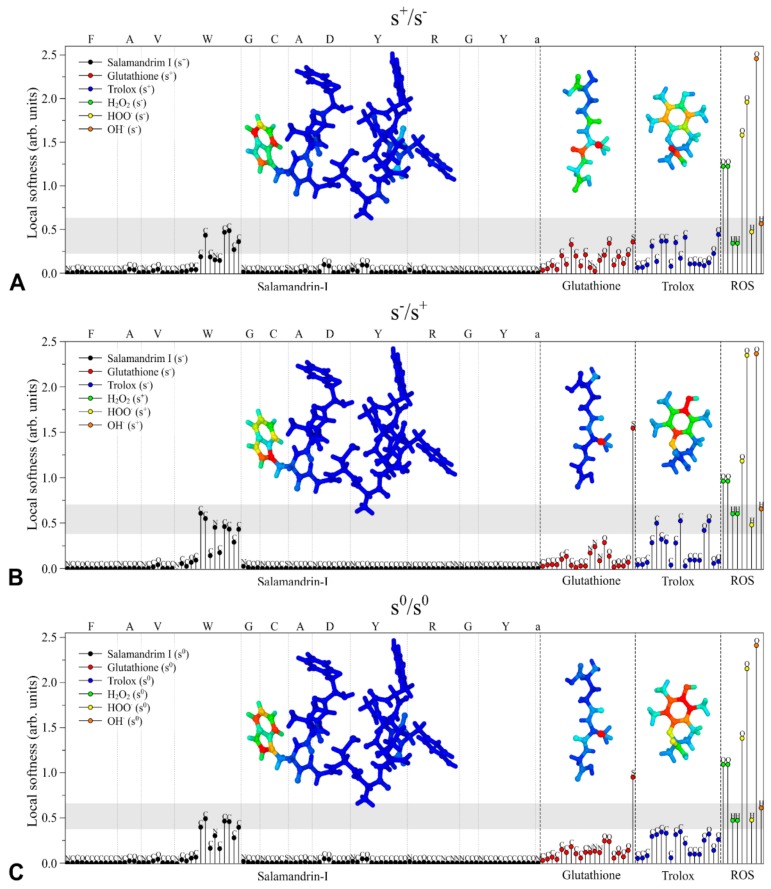
Comparative study of the local chemical softness of salamandrin-I, glutathione, and Trolox in relation to H_2_O_2_, HOO^•^ and OH^•^. Antioxidant activities: (**A**) antioxidants (AOX) as electrophiles (s^+^/s^−^). (**B**) AOX as nucleophiles (s^−^/s^+^), and (**C**) AOX as free-radicals scavengers (s^0^/s^0^). Inset: condensed-to-atom Fukui indexes (CAFI)—red to blue colors define reactive and non-reactive sites.

Figure 5 illustrates the CAFI representation (structures in the inset) and local softness of salamandrin-I, glutathione, Trolox, and common reactive oxygen species (ROS) [35,36]. The colored structures in the inset represent the distribution of the CAFI values on the molecules, where red and blue colors represent, respectively, reactive to non-reactive sites (intermediate values are given using an RGB scale). In general, these indexes indicate which sites are prone to interact with electrophiles (*f*^−^), nucleophiles (*f*^+^), or free radicals (*f*^0^). The stick graphs represent the local softness of the antioxidants (AOX) in relation to the ROS species (H_2_O_2_, HOO^•^ and OH^•^), and according to the hard and soft acids and bases (HSAB) principle, chemical reactions are supposed to occur more effectively between atoms with comparable chemical softness [55]. Once the compounds’ activity was associated with their oxidant/reductive properties, the plausibility was investigated of salamandrin-I, glutathione and Trolox to act as: i) electrophiles (for AOX reduction: s^+^/s^−^), ii) nucleophiles (for AOX oxidation: s^−^/s^+^), and iii) free-radicals (s^0^/s^0^).

It is interesting to note that tryptophan (W) is the only amino acid in the structure of salamandrin-I that presents reactive sites (regions that are not blue) for all the reactions, evidencing that the reactivity of the peptide is centered on this unit, in accordance with other studies that highlight the antioxidant potential of the W unit [56,57]. For glutathione, the reactivity is centered on the sulfur and oxygen atoms, evidencing the well-known reactivity of cysteine amino acid in this structure [58]. The reactive sites are spread over the structure of Trolox.

The gray regions in the graphs highlight the most significative matching between the local softness of the AOXs (salamandrin-I, glutathione, and Trolox) and ROS species. Based on the HSAB principle, salamandrin-I is supposed to present the most effective interaction with ROS for all the cases (s^+^/s^−^, s^−^/s^+^ and s^0^/s^0^), which is mainly associated with tryptophan atoms which have shown higher similarity with the local softness of ROS. Relevant matches are also observed for Trolox carbon atoms. The antioxidant properties of glutathione are apparently associated with the sulfur atom.

It is important to stress that the above presented results do not consider possible changes to the structure and/or composition of the compounds that can occur in the reaction medium. In this sense, additional studies are still necessary to investigate the role of other mechanisms, including the formation of disulfide bonds and other structural effects coming from the interaction with the environment.

### 3.5. Toxicity Studies

Toxicological screening is very important for the development of new drugs and for the extension of the therapeutic usage of existing molecules [59]. We performed several toxicological studies with salamandrin-I: in silico studies, human cell-based experiments, and in vivo assays using a larvae model. Thus, we can have a first assessment of the biosafety of this peptide before future trials of pharmacological applications in specific systems.

The application of computer-based modeling in the search for lead compounds is a promising endeavor in drug discovery, since it often accelerates the process and cuts down costs [60]. Thus, salamandrin-I, glutathione, and Trolox structures (Figure 4A–C) were investigated using in silico toxicological studies with the pkCSM tool [48] (Table 3). Glutathione and Trolox are important well-established antioxidants and are therefore used in this platform for comparison. Together, the toxicity profile of the peptide suggest that salamandrin-I would be well-tolerated by humans and rats, with the values within the recommended range. Likewise, the Ames mutagenicity prediction was negative for the peptide, indicating absence of salamandrin-I-induced mutagenic activity, as well as absence of skin sensitization. The lack of skin sensitivity may be interesting for future applications in dermocosmetic formulations considering its antioxidant potential.

The hepatotoxicity prediction is based on the human liver-related side effects of more than 500 different compounds. The positive result found indicates similarity to a previously described hepatotoxic compound, rather than proof of hepatoxicity itself [48]. Thus, this result indicates further work should include evaluation of the biochemical parameters and enzymes of liver metabolism in vivo in the mammalian models in addition to histopathological analyses.

The blockage of the voltage-dependent ion channel encoded using hERG may affect cardiac repolarization, leading to drug-induced QT interval prolongation, which is a critical side-effect of non-cardiovascular therapeutic agents [61]. The hERG II model is based on the preliminary data of more than 800 compounds, also considering molecular parameters, such as solubility and molecular weight [62]. Our in silico results revealed a positive result for salamandrin-I for hERG II but not for hERG I. Thus, these results suggest a possible salamandrin-I-induced cardiotoxic effect. However, the differences between the two models and the different datasets applied in the same model should be considered. This could explain the variation in the final results of these two models [63].

**Figure 6:**
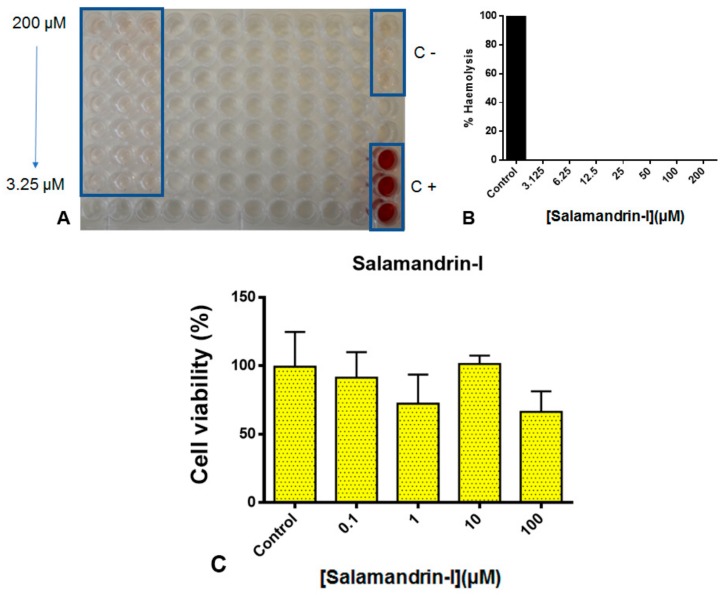
(**A**) Cytotoxicity studies in human microglial cells of salamandrin-I. (**B**) Hemolytic activity of salamandrin-I in human red blood cells/O^+^. Representative 96-well plate image, PBS pH 7.4 (negative control, C−) and Triton X-100 0.1% (positive control, C+). (**C**) Quantitative histogram representation, C+: control (Triton X-100 0.1% represent 100% hemolytic activity). Experiments were performed in triplicate.

In vitro cytotoxicity assays were made, to make preliminary assessment of toxicity, and to enable suitable concentration ranges to be used in future in vivo testing [64]. In this study, there was no statistically significant decrease in cell viability of human microglia treated with salamandrin-I as indicated by the resazurin assay up to 100 µM, suggesting an absence of cytotoxicity for this peptide (Figure 6A). Microglia are a type of glial cells located throughout the brain and spinal cord and account for 10%–15% of all glial cells found within the brain and constantly monitor neuronal functions through direct somatic contacts, and external neuroprotective effects [64]. In vitro non-cytotoxicity at high concentrations suggests the possibility of neuroprotective studies to assess whether the intrinsic antioxidant activity of the molecule can lead to cellular neuroprotection through regulation of microglia activity.

The hemolysis assay is generally considered a rapid and simple, yet worthwhile initial cytotoxicity assay [65]. In general, peptides having high hemolytic activity are not suitable for therapeutic use.

As shown in Figure 6B,C, salamandrin-I has no hemolytic activity to human RBC (O^+^) up to 100 µM. Physicochemical factors such as hydrophobicity, tendency to form linear alpha helix structures, and cationicity may be related to antimicrobial, anticancer, and hemolytic activities [16]. Salamandrin-I presents a random coil structure even in the presence of solvents, such as TFE (Figure 3A). This property may help to increase exposure to specific amino acids, increasing chemical reactivity and electron transfer, but not lead to hemolytic activity in eukaryotic cells. The low molecular weight, presence of tryptophan and tyrosine, and low solubility in aqueous medium (Figure 3B) could suggest that salamandrin-I may come to be internalized in target cells; however, more studies on the mechanism of action, such as fluorescence and/or radiolabeling, need to be conducted to explore these properties.

**Figure 7:**
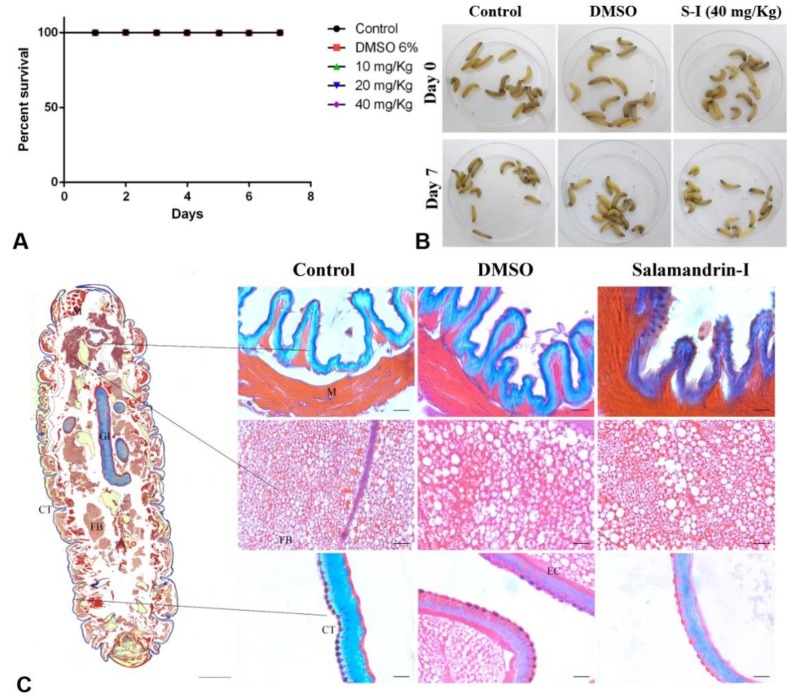
(**A**) *Galleria* *mellonella* log-rank Mantel–Cox survival curve in the presence of different doses of salamandrin-I evaluated for seven days. Note that all data overlap (no deaths were observed). All larvae were injected with 25 μL per gram body weight. Data from two experiments, n = 10 for all groups. (**B**) Representative larvae of groups, 0 and 7 days after treatment, the lack of larvae melanization indicates good tolerance. (**C**) Histological sections of *G. mellonella* (Heidenhain’s aniline blue stain) from control group, DMSO, and salamandrin-I at 40 mg/kg, 7 days after treatment. Whole larva Scale Bar: 1000 µm; Fragment Scale Bar: 20 µm. (FB) fat body; (GI) gastrointestinal tract; (CT) cuticle; (EC) epidermal cell; (M) muscle cell.

Evaluating the toxic effect in vivo using the *G. mellonella* model, revealed that salamandrin-I had no negative effect on the larvae survival. As shown in Figure 7, the larvae treated with doses of 10, 20, and 40 mg/kg remained alive after seven days of monitoring (0% mortality). These results do not differ statistically (log-rank Mantel–Cox) from controls treated with PBS or 6% (*v*/*v*) DMSO. Peptides that have no toxicity in this model have been demonstrated by several studies [66,67,68]. Another way to study the toxic effects in the *G. mellonella* model is through histological analysis [69].

The lack of melanization in larvae 0 and 7 days after treatment demonstrates that the animals were in good physiological condition (Figure 7B). There were no areas of damage or significant histological alterations in the larvae treated with salamandrin-I at 40 mg/kg compared with control larvae (Figure 7C). The histological processing was performed with the whole animal, where the following tissues could be observed in detail: fat body, gastrointestinal tract, cuticle, epidermal cell, and muscle; these are vital tissues for the development of the larva. In the case of muscle tissue, there was a slight change in morphological pattern, suggesting intense muscle activity at a concentration of 40 mg/kg.

These data indicate that an in vivo toxicological study in mammalian models needs to be performed to evaluate the possible myotropic activity of the peptide. Indeed, many peptides in amphibian skins have been described as having myotropic activity, including bradykinin, bombesin, tachykinin, tryptophyllin, caerulein, and cholecystokinin, which are vital for amphibian defensive mechanisms. These related peptides exert contractile effects on isolated ileum and smooth muscles [70]. Considering the global trend to minimize the use of vertebrates in experimentation, there is constant need to develop alternative model systems in vitro, in vivo, or in silico. One such in vivo alternative is the larvae of the greater wax-moth, *G. mellonella* [69].

## 4. Conclusions

This work shows, for the first time, the presence of bioactive peptides in the skin amphibian secretions of the genus Salamandra, specifically using the fire salamander as a model. In addition, salamandrin-I is the first peptide with antioxidant potential found in the Urodela order.

Our experimental and theoretical study indicates that salamandrin-I is a potential antioxidant. Results compared the electronic characteristics of synthetic commercial (Trolox) and endogenous (glutathione) antioxidant molecules, with those of salamandrin-I. These results showed an important balance between the acceptor and donor properties, as well as predicted pharmaceutical parameters for oral administration. Associated with this, salamandrin-I had low toxicity demonstrated by in silico, in vitro, and in vivo models. In addition, the in silico model predicted that, based on the local softness results on the amino acid chain, tryptophan is likely the amino acid responsible for the observed antioxidant activity. Structural similarity with the N-terminal of the CFBD-1 peptide is interesting, especially for antimicrobial activity. Despite this, salamandrin-I did not show antimicrobial activity against Gram-positive or Gram-negative bacteria. However, salamandrin-I has an amidated C-terminal, and to better understand the structure-function relationship with other bioactive peptides of the Salamander species, such as CFBD-1, one will need to determine its complete S-I gene structure using DNA sequencing.

Finally, the presence of antioxidant peptides in the amphibian skin secretions leads to a better understanding about the adaptative physiology of these animals. Indeed, endogenous antioxidants are key for the survival and adaptation of animals to the environment [71]. In addition, it suggests future cellular studies using antioxidant peptides in topical formulations for antiaging pharmaceuticals, or as putative neuroprotectors, which may assist in the development of therapeutic strategies aiming at controlling oxidative stress in neurological disease.

## Figures and Tables

**Table 1:** Salamandrin-I structural similarity with the CFBD-1 sequence. Bold letters represent identical amino acid position.

Peptide	Species	Sequence
CFBD-1	*C. fudingensi*	MAVNGSQGVE**FAVWGCADYRGY**CRAACFAFEYSLGPKGCTEGYVCCVPNTF
Salamandrin-I	*S. salamandra*	**FAVWGCADYRGY**a *

* a (amidation of C-terminal).

**Table 2:** Antioxidant capacities of salamandrin-I peptide compared with reduced glutathione according to different in vitro antioxidant assays. Results are expressed as mg of Trolox equivalent per mg of peptide.

Peptide	In Vitro Antioxidant Activity (Trolox-eq/mg)	
	ABTS Assay	DPPH Assay
Glutathione	1.911 ± 0.003	0.829 ± 0.005
Salamandrin-I	0.285 ± 0.003	0.081 ± 0.005

ABTS (2,2-azino-bis(3-ethylbenzothiazoline-6-sulphonic acid). DPPH (2,2-diphenyl-1-picrylhydrazyl).

**Table 3:** In silico toxicology. Computer-aided toxicity parameters and recommended ranges of oral administration of active compounds.

Model Name	Salamandrin-I	Glutathione	Trolox
Ames toxicity *	No	Yes	No
Max. tolerated dose (human, log mg/kg/day)	0.438	1.104	0.800
hERG I inhibitor	No	No	No
hERG II inhibitor	Yes	No	No
Oral Rat Acute Toxicity (LD_50_, mol/kg)	2.482	2.468	2.382
Oral Rat Chronic Toxicity (LOAEL, log mg/kg bw/day)	10.773	2.919	1.857
Hepatotoxicity	Yes	No	No
Skin sensitization	No	No	No
*T. pyriformis* toxicity (log µg/L)	0.285	0.285	0.197
Minnow toxicity (log mM) ^$^	23.468	4.569	1.674

* The Ames Test combines a bacterial revertant mutation assay with a simulation of mammalian metabolism to produce a highly sensitive test for mutagenic chemicals in the environment. hERG is the human ether-à-go-go related gene; LD_50_ is the median lethal dose; LOAEL is the lowest-observed-adverse-effect level_._^$^ In silico acute toxicity prediction to fathead minnow (*Pimephales promelas*).

## Supplementary Materials

The following are available online at https://www.mdpi.com/2218-273X/10/4/512/s1, Figure S1: Structure prediction of CFBP-1 (*Chinese salamander*) by PEP-FOLD3 program, Table S1: Cartesian coordinates of Salamandrin-I (optimized geometry via DFT), Table S2: Cartesian coordinates of Glutathione (optimized geometry via DFT), Table S3: Cartesian coordinates of Trolox (optimized geometry via DFT).
