# Supplementary material for: The Antioxidant Peptide Salamandrin-I: First Bioactive Peptide Identified from Skin Secretion of Salamandra Genus (Salamandra salamandra)
Source: Biomolecules. 2020 Mar 27;10(4):512. doi: 10.3390/biom10040512 (PMC7226163; doi:10.3390/biom10040512)
Supplement: Supplementary file 1 [file biomolecules-10-00512-s001.pdf]

Article

Salamandrin-I: the first antioxidant peptide identified from skin secretion of the fire salamander (*Salamandra salamandra*)

Plácido et al., 2020.

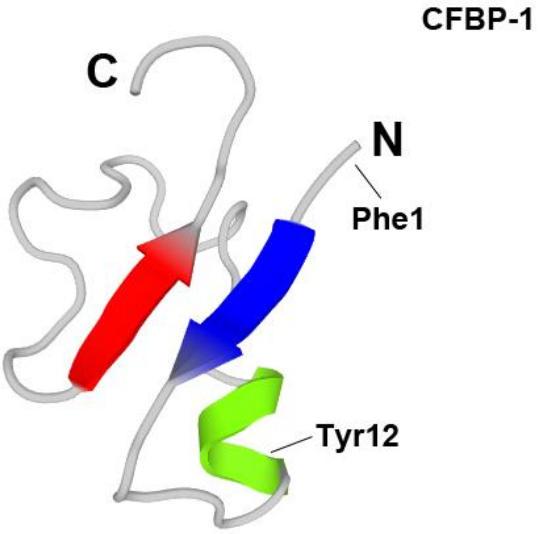

Figure S1: Structure prediction of CFBP-1 (*Chinese salamander*) by PEP-FOLD3 program.

Table S1. Cartesian coordinates of Salamandrin-I (optimized geometry via DFT).

Total number of atoms: 187

Atom	X	Y	Z
N	-1.25418	3.45465	-2.62501
C	-2.30141	3.91561	-1.70899
C	-3.53530	2.99859	-1.79839
O	-4.61083	3.27070	-1.23819
C	-2.67406	5.41504	-1.88152
C	-3.41479	6.06806	-0.73230
C	-4.70894	6.57743	-0.89868
C	-2.79921	6.21681	0.51994
C	-5.37439	7.21020	0.15313
C	-3.46232	6.84257	1.57692
C	-4.75444	7.34274	1.39739
H	-1.92080	3.77532	-0.68919
H	-1.72228	5.93934	-2.02954
H	-3.24436	5.53496	-2.80980
H	-5.20102	6.47354	-1.86200

H	-1.78758	5.84645	0.66985
H	-6.37755	7.59833	0.00015
H	-2.96662	6.94613	2.53807
H	-5.27035	7.83361	2.21743
H	-0.33141	3.75211	-2.30207
H	-1.39418	3.85791	-3.54955
N	-3.34044	1.86236	-2.49697
C	-4.37380	0.84612	-2.68560
C	-4.42051	-0.05710	-1.43335
O	-3.94777	-1.19881	-1.41765
C	-4.11704	0.04517	-3.95605
H	-2.39592	1.73213	-2.85299
H	-5.33077	1.37439	-2.75361
H	-4.07677	0.71536	-4.81811
H	-4.91859	-0.67993	-4.11509
H	-3.17513	-0.50471	-3.88532
N	-5.00333	0.51522	-0.34615
C	-4.80368	-0.05327	0.98685
C	-5.40534	-1.45781	1.13458
O	-4.91789	-2.25651	1.94350
C	-5.31076	0.89220	2.10934
C	-4.44581	2.15978	2.20144
C	-6.80457	1.23343	1.99502
H	-5.20146	1.51298	-0.43008
H	-3.72958	-0.20677	1.14533
H	-5.16236	0.32021	3.03337
H	-3.38896	1.90711	2.33678
H	-4.75934	2.76506	3.05765
H	-4.53162	2.78400	1.30594
H	-7.42964	0.33623	1.97379
H	-7.02329	1.81637	1.09373
H	-7.11348	1.83551	2.85475
N	-6.47253	-1.76773	0.36300
C	-7.11270	-3.07356	0.43181
C	-6.21250	-4.22401	-0.05985
O	-6.49207	-5.38646	0.23647
C	-8.44285	-3.08821	-0.36167
C	-9.44010	-2.05700	0.08650
C	-10.30499	-2.15594	1.15215
C	-9.65905	-0.75198	-0.49124
N	-11.04056	-0.99657	1.27487
C	-10.67048	-0.11400	0.28419
C	-9.11279	-0.05997	-1.59013
C	-11.13094	1.17667	-0.00190
C	-9.56560	1.22331	-1.87492
C	-10.56336	1.83599	-1.08703
H	-6.84162	-1.05821	-0.25672
H	-7.32419	-3.30606	1.47999
H	-8.21933	-2.95449	-1.42690
H	-8.85191	-4.09660	-0.25428
H	-10.45795	-2.97503	1.84081
H	-11.74853	-0.83188	1.97362

H	-8.35639	-0.52250	-2.21936
H	-11.90273	1.64441	0.60198
H	-9.15054	1.76338	-2.72071
H	-10.89747	2.83898	-1.33509
N	-5.16018	-3.87415	-0.84028
C	-4.21917	-4.84051	-1.36436
C	-2.76729	-4.50410	-1.02206
O	-1.87805	-4.65134	-1.87192
H	-4.98330	-2.89236	-1.04185
H	-4.48087	-5.81894	-0.94957
H	-4.26982	-4.90691	-2.45488
N	-2.52213	-4.06890	0.23688
C	-1.26183	-3.39301	0.59046
C	-0.10731	-4.38269	0.91146
O	0.44651	-4.40849	2.01958
C	-1.50708	-2.43554	1.75936
S	-0.32686	-1.01223	1.82117
H	-3.32368	-3.87903	0.83006
H	-0.94222	-2.83801	-0.29871
H	-2.51810	-2.03442	1.69703
H	-1.40323	-2.97298	2.70314
H	0.24094	-1.16606	0.59237
N	0.26646	-5.14573	-0.13504
C	1.37218	-6.09571	-0.11020
C	2.65676	-5.52180	-0.76645
O	3.55152	-6.26375	-1.16860
C	0.97407	-7.42391	-0.75663
H	-0.29410	-5.05072	-0.98781
H	1.61889	-6.26239	0.94410
H	1.82960	-8.10025	-0.76156
H	0.65110	-7.27524	-1.79200
H	0.15495	-7.88353	-0.19880
N	2.72098	-4.16620	-0.79314
C	3.75705	-3.41604	-1.48899
C	4.76581	-2.84664	-0.45613
O	5.26417	-3.59677	0.39848
C	3.08074	-2.37321	-2.41433
C	2.23096	-1.36819	-1.60306
O	2.68595	-0.18682	-1.47623
O	1.18995	-1.81601	-1.05239
H	1.87285	-3.62579	-0.62905
H	4.33111	-4.12932	-2.09052
H	3.83379	-1.85190	-3.00978
H	2.43180	-2.92343	-3.10069
N	5.04287	-1.52860	-0.54857
C	5.91640	-0.78404	0.35202
C	5.06880	0.21598	1.18288
O	5.37199	1.40670	1.29614
C	7.04850	-0.06655	-0.40973
C	8.00782	-1.02167	-1.08465
C	7.86799	-1.35805	-2.43875
C	9.05964	-1.61026	-0.36989

C	8.73935	-2.24976	-3.06080
C	9.94038	-2.50597	-0.97557
C	9.78035	-2.83030	-2.32776
O	10.60819	-3.69777	-2.98455
H	4.46304	-0.95250	-1.15878
H	6.35090	-1.50831	1.04713
H	6.60105	0.60695	-1.14770
H	7.57606	0.56296	0.31197
H	7.06727	-0.90912	-3.02043
H	9.20119	-1.36298	0.67944
H	8.62749	-2.50157	-4.11059
H	10.75215	-2.94696	-0.40245
H	11.28139	-4.02648	-2.37210
N	3.98085	-0.32417	1.78558
C	3.10974	0.38702	2.72321
C	2.42964	1.62208	2.08753
O	2.16429	2.61418	2.76573
C	3.79551	0.76719	4.05280
C	4.67165	-0.31004	4.71011
C	3.98516	-1.55108	5.31121
N	3.32602	-2.45465	4.35337
C	3.93935	-3.18009	3.40871
N	5.27857	-3.25849	3.34946
N	3.22588	-3.84458	2.49602
H	3.74936	-1.28363	1.57532
H	2.29061	-0.30937	2.92855
H	4.42589	1.64179	3.87359
H	3.01017	1.09079	4.74223
H	5.45732	-0.61211	4.01023
H	5.19609	0.16324	5.54806
H	4.71582	-2.12327	5.89415
H	3.20484	-1.24529	6.01085
H	2.31749	-2.50611	4.37849
H	5.85778	-2.72890	3.98002
H	5.69387	-3.56741	2.47611
H	2.20528	-3.87731	2.47830
H	3.72264	-4.26730	1.71939
N	2.08739	1.49916	0.77796
C	1.38784	2.54596	0.06642
C	2.21251	3.27538	-0.99574
O	1.65302	4.02029	-1.80961
H	2.32239	0.67349	0.22034
H	0.51087	2.13756	-0.44487
H	1.04347	3.28720	0.79222
N	3.54757	3.06093	-0.97502
C	4.46844	3.71753	-1.88566
C	4.92419	2.84114	-3.07201
O	5.70258	3.31446	-3.90336
C	5.70385	4.26844	-1.13158
C	5.37120	5.35928	-0.13743
C	5.16484	5.07094	1.22000
C	5.24203	6.69193	-0.55053

C	4.84082	6.07211	2.13374
C	4.91747	7.70585	0.35049
C	4.71563	7.39659	1.70052
O	4.40041	8.34214	2.63739
H	3.93019	2.48111	-0.23519
H	3.92202	4.55137	-2.33633
H	6.19804	3.43423	-0.62024
H	6.39461	4.63800	-1.89351
H	5.26160	4.04654	1.56914
H	5.40172	6.94820	-1.59511
H	4.68564	5.84259	3.18326
H	4.82730	8.73386	0.00826
H	4.35090	9.21020	2.21277
N	4.44094	1.58247	-3.14699
H	3.77215	1.17048	-2.50061
H	4.71642	1.03543	-3.95050

Table S2. Cartesian coordinates of Glutathione (optimized geometry via DFT).

Total number of atoms: 37

Atom	X	Y	Z
S	-1.40860	-1.12685	-2.41350
O	1.20632	2.05303	-0.75918
O	-2.15775	-0.87362	1.06756
O	4.26110	-1.75631	1.50955
O	6.17936	-1.80258	0.33769
O	-5.29037	-0.75882	-1.09051
O	-6.84371	-0.89908	0.54024
N	-0.01928	0.33070	0.06454
N	6.06822	0.83508	-0.27636
N	-3.60381	0.75160	0.39037
C	3.63570	0.57981	-0.22679
C	2.35675	0.40240	0.59739
C	-1.29199	0.83998	-0.40396
C	4.96174	0.29914	0.51789
C	1.14569	1.00848	-0.10411
C	-1.49743	0.65592	-1.92193
C	-2.39061	0.15098	0.42499
C	5.21784	-1.18556	0.74937
C	-4.76194	0.19176	1.04899
C	-5.75213	-0.54043	0.15067
H	3.67505	1.62133	-0.56036
H	3.59786	-0.03368	-1.13415
H	2.17596	-0.64753	0.83643
H	2.46113	0.92882	1.55632
H	-1.32542	1.92180	-0.22490
H	4.88402	0.74264	1.52672
H	-0.07279	-0.48890	0.65808
H	-2.47085	1.05334	-2.21554
H	-0.71479	1.21258	-2.43758
H	6.95134	0.51652	0.11492
H	6.06323	1.84910	-0.19817

H	-3.72318	1.59929	-0.14620
H	-5.31824	0.97191	1.57444
H	-4.41087	-0.52570	1.79553
H	-1.81465	-0.93723	-3.68328
H	4.49861	-2.69399	1.61804
H	-5.98141	-1.24263	-1.57662

Table S3. Cartesian coordinates of Trolox (optimized geometry via DFT).

Total number of atoms: 36

Atom	X	Y	Z
O	1.14350	1.02534	0.67243
O	-3.90081	-0.93983	-0.44899
O	3.67797	-0.78404	-1.13743
O	2.11905	0.67653	-1.85447
C	2.31154	0.21709	0.54733
C	2.06753	-1.14724	1.20458
C	0.88473	-1.86490	0.54863
C	-0.28234	-0.92573	0.30208
C	-0.08860	0.46061	0.37353
C	-1.56707	-1.41692	-0.00157
C	-1.13993	1.37326	0.18039
C	3.43493	1.00014	1.23734
C	-2.43025	0.88171	-0.07397
C	-2.62181	-0.50659	-0.16724
C	2.65709	0.07757	-0.94919
C	-1.84320	-2.89761	-0.15295
C	-0.87975	2.85963	0.25897
C	-3.59753	1.82448	-0.25567
H	1.85627	-0.95627	2.26242
H	2.97096	-1.75867	1.15211
H	0.57416	-2.69581	1.18976
H	1.20916	-2.32159	-0.39707
H	4.38112	0.46089	1.16073
H	3.55062	1.98629	0.77932
H	3.18465	1.13290	2.29294
H	-2.38040	-3.31162	0.71134
H	-2.45142	-3.09901	-1.04328
H	-0.92710	-3.47701	-0.26636
H	0.18125	3.07475	0.13237
H	-1.43785	3.40205	-0.50953
H	-1.18716	3.27301	1.22861
H	-3.54191	2.35547	-1.21473
H	-4.54366	1.28384	-0.23168
H	-3.61794	2.58822	0.52830
H	-3.95445	-1.89502	-0.31748
H	3.87147	-0.80420	-2.09135
